# Calcium Phosphate Modified with Silicon vs. Bovine Hydroxyapatite for Alveolar Ridge Preservation: Densitometric Evaluation, Morphological Changes and Histomorphometric Study

**DOI:** 10.3390/ma14040940

**Published:** 2021-02-17

**Authors:** Guillermo Cadenas-Vacas, Natalia Martínez-Rodríguez, Cristina Barona-Dorado, Luis Sánchez-Labrador, Jorge Cortés-Bretón Brinkmann, Cristina Meniz-García, José María Martínez-González

**Affiliations:** Department of Dental Clinical Specialities, Faculty of Dentistry, Complutense University of Madrid (UCM), Plaza Ramon y Cajal S/N, 28040 Madrid, Spain; gcaden01@ucm.es (G.C.-V.); hospinatmr@hotmail.com (N.M.-R.); cbarona@ucm.es (C.B.-D.); brinkmann55@hotmail.com (J.C.-B.B.); cmenizga@ucm.es (C.M.-G.); jmargo@ucm.es (J.M.M.-G.)

**Keywords:** biomaterials, calcium phosphate, silicon, bovine hydroxyapatite, alveolar preservation

## Abstract

After tooth extraction, the alveolar bone undergoes a physiological resorption that may compromise the future placement of the implant in its ideal position. This study evaluated bone density, morphological changes, and histomorphometric results undergone by alveolar bone after applying a new biomaterial composed of calcium phosphate modified with silicon (CAPO-Si) compared with hydroxyapatite of bovine origin (BHA). Alveolar ridge preservation (ARP) was performed in 24 alveoli, divided into a test group filled with CAPO-Si and a control group filled with BHA. Three months later, the mineral bone density obtained by the biomaterials, horizontal and vertical bone loss, the degree of alveolar corticalization, and histomorphometric results were evaluated. Both biomaterials presented similar behavior in terms of densitometric results, vertical bone loss, and degree of alveolar corticalization. Alveoli treated with CAPO-Si showed less horizontal bone loss in comparison with alveoli treated with BHA (0.99 ± 0.2 mm vs. 1.3 ± 0.3 mm), with statistically significant difference (*p* = 0.017). Histomorphometric results showed greater bone neoformation in the test group than the control group (23 ± 15% vs. 11 ± 7%) (*p* = 0.039) and less residual biomaterial (5 ± 10% vs. 17 ± 13%) (*p* = 0.043) with statistically significant differences. In conclusion, the ARP technique obtains better results with CAPO-Si than with BHA.

## 1. Introduction

Rehabilitation by means of dental implants is an everyday procedure employed to restore lost teeth. When inserting a dental implant, sufficient bone volume is necessary to permit ideal implant placement in all three spatial dimensions [1]. However, the loss of a tooth from the alveolus triggers a cascade of biological events that lead to an irreversible reduction of the alveolar bone crest in both horizontal and vertical direction [2,3,4].

A systematic review conducted by Tan et al. [5], which evaluated the dimensional changes suffered by the alveolus after dental extraction, observed horizontal bone losses of 29–63% and vertical losses of 11–22% six months after extraction. Andrés-Veiga et al. [6] reported that these bone losses varied from one individual to another and may differ in the same subject at different times of life due to the influence of local and systemic factors.

The reduction in bone volume is due to a lack of stimulation to the residual bone, which triggers reductions in trabecular bone and in bone density [7]. Bone vascularization will also be altered by resorption, changing from intraosseous to periosteal centripetal irrigation [8]. All these changes will condition the potential for ideal implant placement in a three-dimensionally correct position [2,3,4].

Alveolar ridge preservation (ARP) is a surgical procedure, which has been shown to significantly decrease the collapse of the alveolar bone ridge after dental extraction. In ARP, a biomaterial is placed in the alveolus in order to facilitate subsequent rehabilitation by an implant [9,10,11]. A meta-analysis of the ARP technique conducted by Willenbacher et al. [4] concluded that 20.8% of non-preserved alveoli require treatment by an additional bone graft for implant rehabilitation, while only 9.9% of preserved alveoli require grafting. Actually, there is not enough scientific evidence to determine which alveolar filling material is superior in this technique [12,13].

Among the bone substitutes available for ARP or for grafting, autologous bone is the only bone material that offers osteoinductive, osteoconductive, and osteogenic properties [14]. However, this option suffers several disadvantages, the main ones being the need for a second surgical site, increased morbidity at the donor site, limited availability of bone extracted intraorally, reports of unpredictable resorption, and the need for general anesthesia for the harvesting of extraoral bone. For these drawbacks, bone alternatives are thought to replace autologous bone and its properties in bone regeneration [15,16].

Allogenic grafts were introduced as an alternative to autologous bone but suffer the risk of transmitting diseases from the donor [15], although it is reported to be extremely low [17].

Among xenogeneic materials hydroxyapatite of bovine origin (BHA) is the most studied and has been used successfully in several investigations. This material has succeeded in preserving the dimensions of the bone crest after tooth extraction [18,19,20].

In the last decades, an expansion has been carried out in the search for new synthetic biomaterials that might successfully replace advantages of autologous bone [14,15]. Among synthetic materials, those composed of calcium phosphate are widely used in the scientific literature and have shown promising results in different bone regeneration studies, being used successfully in techniques such as sinus lift, horizontal augmentation of the bone crest, or in the ARP [21,22,23,24,25].

Any biomaterial used as a bone substitute must remain in its original state only temporarily, acting as a support for bone neoformation. In this way, it must eventually undergo complete resorption, gradually replaced through the neoformation of functional bone tissue; the sooner this process is completed, the sooner the implant can be inserted [1]. Biomaterial resorption is of great importance as successful bone neoformation will make it possible for the implant to osteointegrate and achieve optimal bone-to-implant contact. Osteointegration can only take place between functional bone tissue and the implant surface, not between residual biomaterial and the implant surface [26].

Although different quantities of residual graft can difficult the osseointegration process, it has been seen that even with granules remaining in the surrounding of the implant, a normal bone-implant interface is produced at the histological view since the granules participate in the remodeling process [27]. A study by Crespi et al. [27] evaluated the behavior of implants placed in alveoli in which ARP had been performed with three biomaterials of different composition (magnesium-enriched hydroxyapatite, calcium sulfate, and BHA), revealing that there were no significant differences in the results between the groups, obtaining all of them a 100% survival rate at 24 months.

Moreover, when oral surgeries that generate bone defects with complicated shapes are performed, they must be filled by a biomaterial, so in this context, biomaterials in the form of granules are more useful than materials in block form [28].

This situation has motivated the development of new biomaterials. These are composed of alkaline calcium phosphate, which is resorbed quickly and enjoys a balance between high solubility and resorption. In addition, calcium phosphate stimulates bone neoformation [26], as has been proved when applicated to dental implant surfaces, promoting the expression of osteoblastic differentiation markers and improving substantial osteoinduction [29]. These new materials with alkaline calcium phosphate are considered excellent bone substitutes due to their capacity for forming a union with bone and stimulating bone maturation [26].

In the last years, researchers have investigated the application of different substances to bone grafts to improve their properties. Stem cells of different origins are a possible source of alveolar bone regeneration. Among the most suitable cells for bone regeneration are embryonic stem cells, mesenchymal stem cells and induced pluripotent stem cells. Furthermore, the combination of growth factors together with suitable biomaterials can increase the osteogenic potential of mesenchymal stem cells. [30]. On the other hand, metallic ions combined to materials can improve angiogenesis and osteogenesis [31].

With the objective of achieving the greatest bone formation in the shortest possible time, copious research has focused on improving the osteogenic capacity of calcium phosphate-based bone graft materials. The ultimate aims are to use these biomaterials for implant-based rehabilitations and to reduce treatment time. In this context, various authors have proposed incorporating silicon (Si) into biomaterials to improve their osteogenic properties [32,33,34,35,36,37,38].

Si is considered necessary for adequate bone and cartilage development and growth and it has been noted that synthetic biomaterials based on calcium phosphate that include some level of Si in their structure present better biological performance. The improvement in performance is attributed to the fact that Si stimulates osteoblast activity and bone formation [35]. Moreover, several studies have demonstrated that incorporating Si increases angiogenesis, favoring bone neoformation [39,40]. This was seen in a study by Patel et al. [36], which performed histomorphometric quantification of neoformed bone comparing two composites: one consisting of hydroxyapatite the other consisting of hydroxyapatite modified with Si. The results revealed that the Si-modified material obtained 15.5% more bone growth and 12.7% additional implant surface coverage in comparison with the material without Si.

The aim of this study was to evaluate the bone density obtained, morphological changes to the alveolar crest, and histomorphometric results of alveoli treated by means of the ARP procedure with a new biomaterial composed of calcium phosphate modified with Si (CAPO-Si) compared with BHA.

## 2. Materials and Methods

### 2.1. Study Design and Participants

A randomized split-mouth clinical trial was carried out among patients with uniradicular and biradicular teeth requiring extraction. The patients were recruited from the Oral Surgery Service of the Faculty of Dentistry at the Complutense University of Madrid (UCM). Two alveolar sites were established in each patient: A test alveolus filled after dental extraction with CAPO-Si and a control alveolus filled with BHA. All subjects gave their informed consent for inclusion before they participated in the study. The study was conducted in accordance with the Declaration of Helsinki, and the protocol was approved by the Ethics Committee for Clinical Trials at the San Carlos Hospital, Madrid (Spain) (Reg. N° 20/594-EC, 23 September 2020).

A total of 12 patients, presenting indications for bilateral tooth extraction, were selected from the Oral Surgery Service of the Faculty of Dentistry at the UCM. The main researcher (G.C.-V.) evaluated the patients who were provided with all the information about the purpose, the procedures involved, and the risks and benefits of participating in the trial. All patients signed an informed consent form.

Inclusion and exclusion criteria were as follows:

#### 2.1.1. Inclusion Criteria

- Patients aged over 18 years.
- Patients with uniradicular or biradicular teeth requiring bilateral extraction for various reasons (caries, trauma, crown fracture, root fracture, etc.).

#### 2.1.2. Exclusion Criteria

- Smokers.
- Upper molars, lower molars, and lower incisors were excluded.
- Patients presenting cortical defects in the alveolus, whether pre-operative or as a result of dental extraction.
- Presence of acute or chronic alveolar infection.
- Patients with calcium disorders or immunodeprived patients.
- Patients in treatment by bisphosphonates (oral or injected), corticosteroids, immunosuppressants, radiotherapy, or drugs that would interfere with calcium metabolism.

The trial included 12 patients who were intervened bilaterally (24 extractions). On the control side BHA was used as graft material after extraction. On the test side CAPO-Si was used as graft material after extraction.

Sample size was determined by a pilot study was carried out at the Oral Surgery Service of the Faculty of Dentistry at the UCM based on the primary outcome parameter (new bone formation). The study, with a split-mouth design, enrolled 3 patients (6 alveoli) obtaining a mean of new bone formation of 21 ± 7% on the test side and 13 ± 5% on the control side. Using G Power 3.1 (Dusseldorf, Germany) the estimation resulted in 9 alveoli per group considering an alpha-type error of 0.05 and a beta-type error of 0.20. To avoid possible dropouts of patients during the study the number of patients was increased to 12.

All surgeries were performed bilaterally by a single blinded clinician (G.C.-V.). The patients were also blinded. Randomization was performed using opaque envelopes whose content determined group assignation as control or test side by another researcher (J.M.M.-G.).

### 2.2. Bone Graft Biomaterials Tested

Control alveoli were filled with deproteinized BHA (Bio-Oss^®^, Geistlich Biomaterials, Wolhuser, Switzerland). Bio-Oss^®^ is a low crystalline apatite with a 7% content of carbonate. It has a calcium quantity of 37.1 ± 0.7% and a phosphorous quantity of 17.8 ± 0.5%. The BHA particle size was 0.25–1 mm with similar porosity to human bone (75–80%). 

Test alveoli were filled with a porous glass ceramic, composed of alkaline calcium orthophosphate, formula Ca_2_KNa(PO_4_)_2_ (Osseolive^®^, Curasan AG, Kleinostheim, Germany). Its composition included Ca (22.5 ± 2%), PO_4_ (48.4 ± 3%), Na_2_O (9.6 ± 2%), K_2_O (13.7 ± 2%), MgO (2.8 ± 2%) and SiO_2_ (3.0 ± 1%). The ceramic contains a 4% aggregate of sodium magnesium silicate. The size of the biomaterial particles was 0.25–1 mm with a porosity of 75 ± 5%. Both study alveoli also received resorbable porcine collagen membranes sized 25 × 30 mm (Osgide^®^, Curasan AG, Kleinostheim, Germany).

### 2.3. Alveolar Preservation Surgery

**Figure 1:**
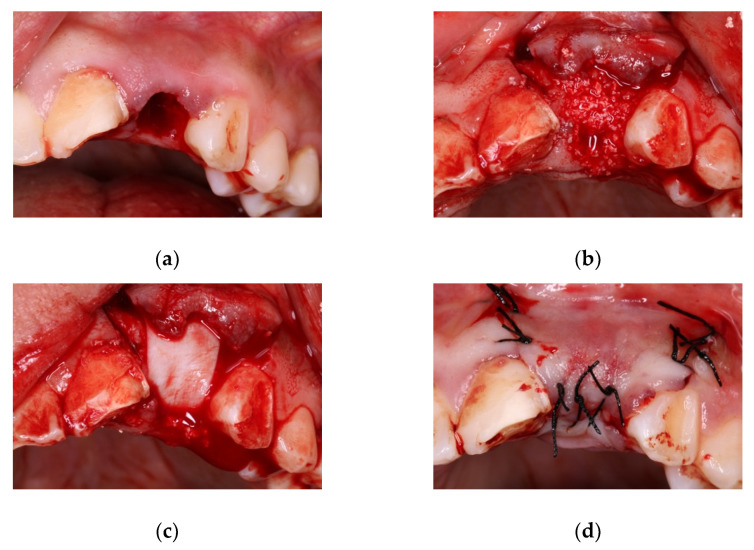
(**a**) Alveolus at extracted tooth site; (**b**) filling the alveolus with biomaterial; (**c**) placement of resorbable collagen membrane; (**d**) flap closure with sutures.

Firstly, the surgical field was disinfected with chlorhexidine 0.12% (Clorhexidina Lacer^®^, Lacer SA, Barcelona, Spain), and then infiltrated with 40/0.01 mg/mL articaine with epinephrine as local anesthetic (Ultracain^®^, Laboratorios Normon SA, Madrid, Spain). Dental extraction was performed as atraumatically as possible, checking the integrity of cortical bone and curetting the alveolus rigorously (Figure 1a).

Afterwards, a full thickness flap was raised and the alveolus was filled with one of the biomaterials (Figure 1b), placing a resorbable collagen membrane sized 25 × 30 mm (Osgide^®^, Curasan AG, Kleinostheim, Germany) (Figure 1c). The flap was then replaced and sutured (size 4-0) (Figure 1d).

Post-operative measures consisted of anti-inflammatory treatment (100 mg diclofenac sodium every 12 h for 14 days) and antiseptic mouthwash with chlorhexidine 0.12%. Antibiotic coverage was just administered in case of postoperative infection. Patients returned to the clinic 7 days later for suture removal. Check-up appointments were scheduled during the first and second months post-surgery, when periapical radiographs were taken to monitor correct tissue healing.

### 2.4. Evaluation of Outcomes

#### 2.4.1. Densitometric Analysis

**Figure 2:**
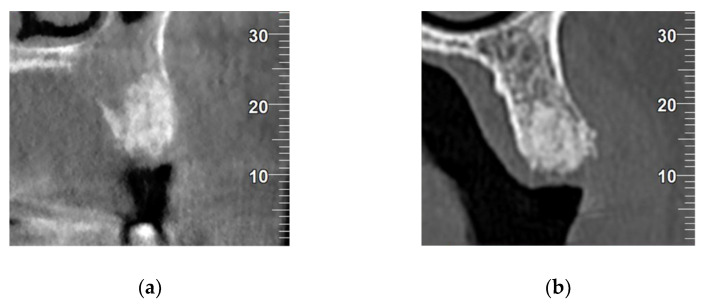
(**a**) Coronal section of control biomaterial (BHA); (**b**) coronal section of test biomaterial (CAPO-Si).

A Cone-Beam Computed Tomography (CBCT) of all patients were captured 3 months after extractions (Newtom model 5G XL, Verona, Italy) to evaluate the bone density obtained by each biomaterial. Densitometric analysis was performed using NNT Viewer 7.2 software, expressing density in the biomaterial graft area in Hounsfield units (HU). Figure 2 provides a radiographic comparison of the control and test biomaterials.

#### 2.4.2. Analysis of Bone Loss

Vertical alveolar bone was measured from the buccal side of the alveolar bone crest in the middle of the alveolar socket at the moment of dental extraction by fabricating an acrylic splint supported by the teeth adjacent to the extraction site; the splint had an insertion orifice, allowing measurement with a calibrated periodontal probe (Hu-Friedy^®^, Chicago, IL, USA.). The total width was measured as the distance between the most coronal portions of the buccal and the palatal/lingual bone crest in the center of the socket with a caliper (Medesy^®^, Maniago, Italy).

The same measurements were repeated 3 months after extraction. Vertical and horizontal bone losses were calculated by subtracting initial values from the values obtained at 3 months.

#### 2.4.3. Evaluation of Degree of Alveolar Crest Corticalization

**Figure 3:**
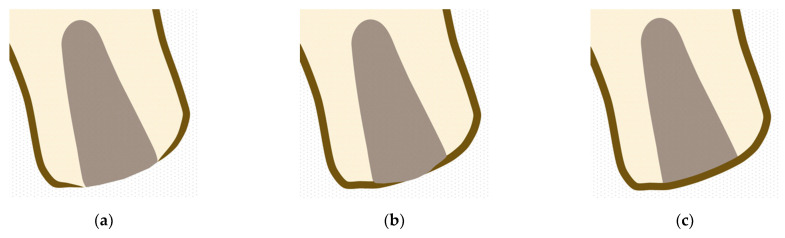
(**a**) Corticalization absent at crestal level; (**b**) partial corticalization at crestal level; (**c**) complete corticalization at crestal level.

This evaluation was carried out using CBCTs captured 3 months after extraction. This was expressed as one of three degrees of corticalization:

- Absent: When no union was observed between vestibular and palatine/lingual cortical at alveolar crestal level (Figure 3a).
- Partial: When the union between vestibular and palatine/lingual cortical was interrupted at alveolar crestal level (Figure 3b).
- Complete: When union was observed between vestibular and palatine/lingual cortical bone at alveolar crestal level (Figure 3c).

#### 2.4.4. Histomorphometric Analyses

After 3 months follow-up, dental implants were placed in the preserved sites. A sample of the biomaterial was collected for histomorphometric analysis. Samples were obtained from patients using a bone trephine connected to a surgical motor at low speed and continuous irrigation, in a corono-apical direction being performed in the center of the alveolus. Samples were fixed in buffered 10% formaldehyde solution for at least 2 weeks before processing.

Trephines with the tissues were dehydrated through a graded ethanol series (70–100%) and infiltrated with 4 graded mixtures of ethanol and an infiltrating resin, glycomethacrylate (Technovit 7200 VLC^®^, Heraus Kulzer GMBH, Werheim, Germany). The last two infiltrations were done with the pure infiltrating resin under vacuum. The samples were then polymerized, first under low-intensity UV light for 4 h and then under high-intensity UV light for 12 h. Lastly, the samples were introduced in an oven at 37 °C for 24 h to ensure complete polymerization.

The samples were prepared and then mechanically micropolished (Exakt Apparatebau, Norderstedt, Germany) with 1200 and 4000 grit silicon carbide papers (Struers, Copenhagen, Denmark) to get samples approximately 50 μm thick. The slides were stained with Laczkó-Lévai stain for histological examination and histomorphometric analysis.

The composition of Laczkó-Lévai staining solution was methylene blue and azure II (each 0.25% in a 0.5% solution of sodium carbonate in distilled water), 0.5% basic fuschsin, and 0.5% sodium carbonate in distilled water. After staining, samples are rinsed in tap water and if it is required, stain precipitates are cleaned with an acetone-soaked tissue. The samples are meticulously dried with a soft cloth. With this method, samples can be examined at a wide range of magnifications and three-dimensional character can best be appreciated at magnifications [41].

All sections were analyzed using light microscopy and a PC-based image capture system (BX51, DP71, Olympus Corporation, Tokyo, Japan) and histometrically observed with a magnification of ×100. A calibrated independent examiner blinded to treatment group assignation took the measurements. The proportions of each defect occupied by bone, biomaterials, and soft tissue were recognized from the digital histological images using a pen computer (Cintiq Companion, Wacom, Düsseldorf, Germany), were then colored (Photoshop, Adobe, San José, CA, USA) and digitally measured with an automated image analysis system (CellSens, Olympus Corporation). To avoid results alterations, as in each trephine, we obtained 2 sections, and we calculated the mean of the results obtained of both slides.

### 2.5. Statistical Analysis

The data collection was entered in Excel spreadsheet (MS Excel 2007, Microsoft Inc., Redmond, WA, USA). An independent statistician analyzed the data with statistical software (SPSS, version 17.0, Chicago, IL, USA).

This clinical trial compared CAPO-Si vs. BHA as grafts materials, whose effects were evaluated in terms of new bone formation, lamellar bone, residual biomaterial, connective tissue, radiographic bone density, horizontal bone loss, vertical bone loss, and degree of corticalization.

For all variables, descriptive statistics were calculated (mean, standard deviation, and frequency). Quantitative variables (new bone formation, lamellar bone, residual biomaterial, connective tissue, radiographic bone density, horizontal and vertical bone loss) were evaluated with Student’s T-test. Qualitative variables were evaluated with the chi-squared test. 

A 95% confidence interval was used for all results (significance level *p* < 0.05).

## 3. Results

A total of 12 patients underwent bilateral surgery (24 extractions). Twelve post-extraction alveoli were treated with BHA and twelve with CAPO-Si. The average age of subjects was 51 ± 10 years. The sample comprised seven women (58.33%) and five men (41.67%). The study’s follow-up took place three months after dental extraction; all patients completed the follow-up period.

With regard to clinical outcomes, primary flap closure was achieved in all cases, without any complications, infections or membrane exposures during the follow-up period.

### 3.1. Densitometric Results

In alveoli treated with BHA, mean mineral bone density was 1076 ± 124 HU, three months after dental extraction. 

Mean mineral bone density obtained in alveoli treated with CAPO-Si 3 months after dental extraction was 1118 ± 113 HU.

No statistically significant differences in bone density were found between test and control alveoli. No significant relationships were found between patient age and the bone density generated by the biomaterials.

### 3.2. Bone Loss Results

**Figure 4:**
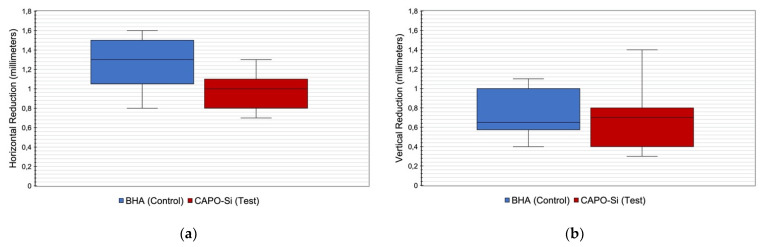
(**a**) Comparison of horizontal bone loss obtained in control and test alveoli with a lower horizontal bone loss in the calcium phosphate modified with silicon (CAPO-Si) group in comparison to the hydroxyapatite of bovine origin (BHA) group three months after ARP techniques (*p* = 0.017); (**b**) comparison of vertical bone loss obtained in control and test alveoli showing a similar vertical bone loss between both groups three months after ARP techniques with no significant differences between groups (*p* = 0.299).

Three months after dental extraction, mean horizontal crestal bone loss in control group alveoli was 1.3 ± 0.3 mm, while in the test group, it was 0.99 ± 0.2 mm with statistically significant difference (*p* = 0.017). Figure 4a shows a comparison of horizontal bone loss between control and test alveoli.

Mean vertical bone loss three months after dental extraction was 0.7 ± 0.2 mm in alveoli treated with BHA (control) and 0.7 ± 0.3 mm in alveoli treated with CAPO-Si (test) without significant difference. Although vertical bone loss was greater in older control subjects, no significant relation was found between age and vertical bone loss (*p* = 0.299). Figure 4b shows a comparison of vertical bone loss between control and test alveoli.

The horizontal and vertical dimensional changes of the test and control group three months after dental extraction are shown in Table 1.

### 3.3. Degree of Corticalization of Crestal Bone

Two control alveoli (16.66%) presented no corticalization (absent), five (41.67%) partial corticalization, and five (41.67%) complete corticalization. 

Among test alveoli, two (16.66%) showed no corticalization (absent), three (25%) partial corticalization, and seven (58.34%) complete corticalization.

No statistically significant differences in the degree of corticalization were found between control and test alveoli. No significant relations were found between gender and the degree of corticalization obtained in control and test alveoli. Table 2 shows the distribution of different degrees of corticalization obtained in control and test alveoli.

### 3.4. Histomorphometric Results

In general, samples consisted of a portion of bone composed of a lamellar area surrounded by bone at different stages of maturation (immature and remodeling bone). Adjoining this region, another area was observed composed of biomaterial with a variable proportion of bone. Signs of necrosis or infection were not present.

A larger quantity of neoformed bone was found in the test group than the control group (23 ± 15% vs. 11 ± 7%, respectively) with statistically significant difference (*p* = 0.039). The amount of lamellar bone was also greater in the test group than the control but without statistical significance (14 ± 10% vs. 9 ± 10%, respectively).

Regarding the quantities of residual biomaterial, this was smaller in the test group than the control (5 ± 10% vs. 17 ± 13%, respectively) with significant difference (*p* = 0.043). Connective tissue amount was also smaller in the test group than the control although without statistical significance (58 ± 16% vs. 62 ± 7%, respectively). Table 3 compares the quantities of neoformed bone, lamellar bone, residual biomaterial, and connective tissue between the two groups.

**Figure 5:**
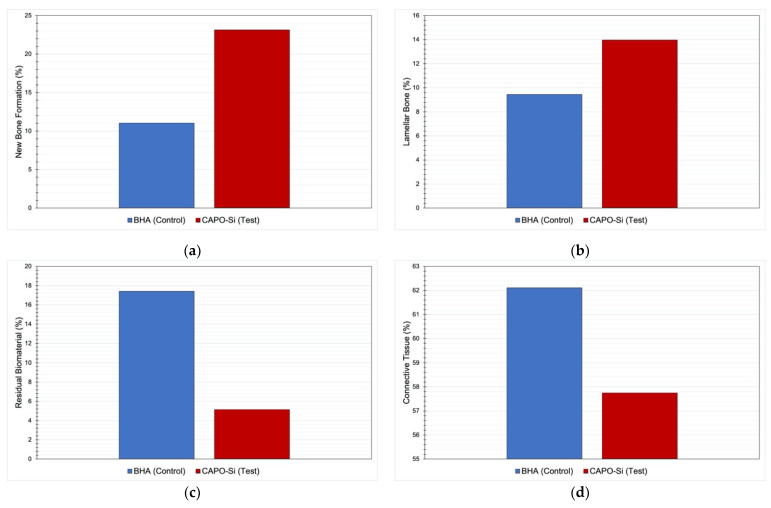
(**a**) Comparison of new bone formation in control and test alveoli with a significant bone neoformation in CAPO-Si group when compared to the BHA group (*p* = 0.039); (**b**) comparison of lamellar bone in control and test alveoli with a greater lamellar bone formation in the test group than the control, but without statistical significance (*p* = 0.342); (**c**) comparison of residual biomaterial in control and test alveoli being smaller in the test group than the control with significant difference (*p* = 0.043); (**d**) Comparison of connective tissue in control and test alveoli with a lower connective tissue in the test group than the control, but without statistical significance (*p* = 0.454).

Figure 5a shows a comparison of the quantities of neoformed bone between the two groups, while Figure 5b compares lamellar bone, Figure 5c residual biomaterial, and Figure 5d connective tissue.

**Figure 6:**
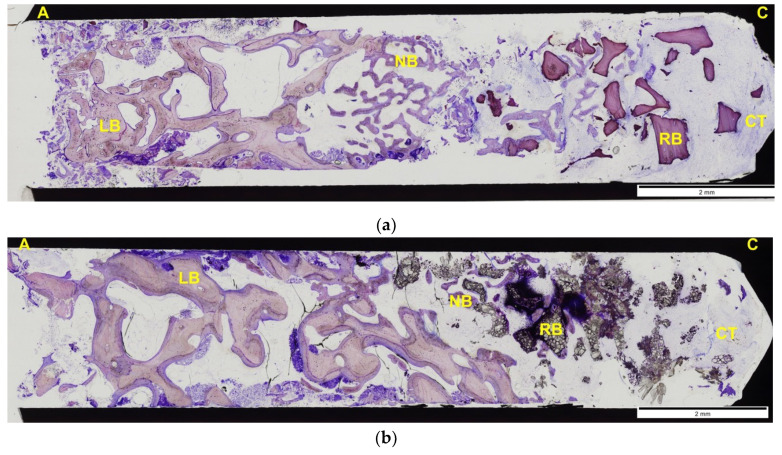
(**a**) Histological section of alveolus treated with BHA (control group). Immature bone formation in the vicinity of the lamellar bone, forming trabeculae to fill the defect with some degree of lamellar bone remodeling. The biomaterial granules are mostly surrounded by dense connective tissue, although in some areas the formation of bone trabeculae is observed with some direct contact with the material, although scarce; (**b**) histological section of alveolus treated with CAPO-Si (test group). The edges of the defect show obvious signs of remodeling with lamellar bone formation. Inside the defect, immature bone formation is observed in close contact with the biomaterial particles, which penetrates between them. In the coronal portion, the material appears surrounded by dense connective tissue and remains of epithelial component. NB: Neoformed Bone; LB: Lamellar Bone; RB: Residual Biomaterial; CT: Connective Tissue; A: Apical Orientation; C: Crestal Orientation.

Figure 6a shows a histological section of an alveolus treated with BHA; Figure 6b shows a histological section of an alveolus treated with CAPO-Si.

## 4. Discussion

In the present study, a modified biomaterial composed of CAPO-Si with the trade name Osseolive^®^, was evaluated in the clinical context of the ARP technique by means of densitometry measured in HU, morphological changes to the alveolar bone crest, and histomorphometric results, comparing CAPO-Si with BHA.

The CAPO-Si material had already been shown to possess excellent osteogenic properties in clinical and preclinical research. In this context, an in vitro study by Knabe et al. (2008) [1] compared three biomaterials used as bone substitutes. The composite that was the subsequent basis for Osseolive^®^ obtained greater stimulation of osteoblast phenotype expression than a beta-tricalcium phosphate (ß-TCP) composite, presenting a greater capacity for osteoblast differentiation. The particles of this biomaterial showed a high resorption rate due to various particle degradation processes, showing itself to be adequate for guided bone regeneration offering a good balance between resorption and osteogenesis.

Osteogenic differentiation is defined by three main biological phases: Cell proliferation, cell maturation, and matrix mineralization [26,28]. These stages are characterized by the expression of specific osteogenic markers. Alkaline phosphatase (ALP) is expressed during the post-proliferative period of extracellular matrix maturation [28]. On this pretext, an in vitro study by Bernhardt et al. (2013) [28] of Osseolive^®^ found that this biomaterial presented correct cytocompatability and osteogenic marker expression. Osteoblast cells were able to attach to and proliferate on the material’s granules, allowing osteogenic differentiation and it was found to show significantly greater ALP activity in osteoblast cells compared with ß-TCP samples. 

Another in vivo study by Knabe et al. (2019) [26] performed in sheep shoulder blades compared Osseolive^®^ with a ß-TCP composite containing 4% sodium magnesium silicate, and with pure ß-TCP as control material. The three materials presented significant differences in biodegradability, Osseolive^®^ showing the greatest biodegradability over the study period, followed by pure ß-TCP and finally the doped ß-TCP. Osseolive^®^ had a stimulating effect on bone formation and osteogenic marker expression, producing faster bone regeneration in critical size bone defects.

A clinical study of Osseolive^®^ carried out by Knabe et al. (2017) [42] reported significantly more bone formation and better histological outcomes six months after treating maxillary sinus lift showing a higher proportion of contact between bone and biomaterial, and greater biomaterial particle degradation in the 19 patients treated with Osseolive^®^, compared with 19 who received ß-TCP graft. In addition, samples treated with Osseolive^®^ obtained greater specific antibody expression of type I collagen (main component of the mineralized matrix), osteocalcin and bone sialoprotein.

On the other hand, the study by Crespi et al. [43] compared alveolar filling with BHA against an unfilled control group. The results of the biopsies collected at four months showed an inclusion of BHA particles in bone tissue generating a thick tissue network in which the biomaterial granules were completely surrounded by vital bone. In addition, the group in which the biomaterial was used showed an increase in the expression of ALP, type I collagen and osteopontin, thus showing a higher potential in the mRNA encoding for type I collagen, higher metabolic activity to generate mineralized matrix, and a lower differentiated osteoblast phenotype.

HU evaluated from CBCTs are used to express bone quality and density. HUs place a value on a material’s radiological density, whereby the radiodensity of distilled water at standard temperature and pressure (STP) is defined as zero HU, while the radiodensity of air at STP is defined as −1000 HU. Most bone density values vary between 100 and 1900 HU [44].

Misch [45] established a classification system for assessing bone quality on the basis of HU, distinguishing the following bone densities:

- D1: Dense cortical bone: >1250 HU.
- D2: Dense to porous cortical bone and thick trabeculae: 850–1250 HU.
- D3: Thin porous cortical bone and fine trabeculae: 350–850 HU.
- D4: Fine trabeculae: 150–350 HU.
- D5: Bone with incomplete mineralization: <150 HU.

The results of the present study showed that the biomaterial’s mean mineral density in alveoli treated with CAPO-Si was greater than alveoli treated with BHA, being 1118 HU and 1076 HU, respectively. Mean density in both biomaterials corresponded to type D2 bone according to Misch’s classification. Both biomaterials presented similar densitometric behavior since no statistically significant difference was found between the mean values obtained.

These results are similar to those obtained by Henao et al. (2016) [46] who compared the use of two biomaterials used for ARP. The test group was filled with β-TCP with chitosan, while the control group was filled with a biphasic material composed of hydroxyapatite and β-TCP. HU evaluation obtained from CBCTs after three months were used to evaluate bone density and quality in 37 alveoli, obtaining mean densities of 1052 HU in the β-TCP group and 1020 HU in the biphasic group. It was concluded that there were no significant differences among the biomaterials. 

In another recent study by Del Canto-Díaz et al. (2019) [47] of ARP used fresh dentin with demineralization as filling material, expressing densitometry in HU evaluated from CBCTs. Alveoli treated with dentin presented less vertical and horizontal cortical resorption, and higher densitometry values 16 weeks after ARP than alveoli that had not been filled with any biomaterial, obtaining 922.68 HU and 564.35 HU, respectively.

Regarding the bone loss values obtained in the present study, alveoli treated with CAPO-Si showed less horizontal bone loss in comparison with alveoli treated with BHA, with statistically significant difference; these results concur with the literature. 

A study by Barone et al. [48], with a three-month follow-up period, obtained a horizontal bone loss of 1.33 mm in a group treated with porcine cortical bone and 0.93 mm in a group treated with porcine spongy cortical bone, while control group alveoli were not filled with any material and presented a mean loss of 3.60 mm.

In the present study, vertical bone loss was less in alveoli treated with CAPO-Si, although the difference did not reach statistical significance. 

A study by Aimetti et al. [49] found a vertical loss of 0.5 mm after three months in a test group treated with a calcium sulfate, while control group alveoli were unfilled by any biomaterial, obtaining a vertical bone loss of 1.2 mm. The results showed statistically significant difference.

The ARP technique through the use of biomaterials in immunosuppressed patients, in order to preserve the width and height dimensions of the bone crest, could be a common procedure that would help to prevent the future need of complex surgical techniques that increase the morbidity and risk of complications for implant rehabilitation procedures in this type of patients. Furthermore, it must be taken into account that survival rates in implantology in immunosuppressed patients appear to be related to adequate patient selection, meticulous surgical technique, rigorous follow-up, and appropriate antimicrobial protocol [50].

In untreated alveoli, corticalization or alveolar closure takes place between the fourth and eighth week after dental extraction, as reported by Araújo and Lindhe [51]. Corticalization is due to the formation of a bone bridge at coronal level that joins the lingual and vestibular cortical bone [51]. Similar results were obtained in a study by Scala et al. [52], who observed the formation of a bone bridge 30 days after dental extraction; this bone bridge had developed into mature bone within 90 days.

As the present study found, the natural process of alveolar corticalization is altered when the alveoli are filled with a biomaterial. Ninety days after dental extraction, the bone bridge that develops connecting vestibular and palatine/lingual cortical bone at coronal level was not completely formed in all cases. In this sense, the present study was innovative in that it distinguished between different degrees of corticalization in alveoli treated with ARP by means of a crestal bone classification system.

As for the histomorphometric results in the present study, the test group obtained a larger quantity of neoformed bone (23%) than the control group (11%) with a statistically significant difference. Moreover, the mean percentage of residual biomaterial was significantly lower in the test group (5%) than the control group (17%). These results point to a faster resorption rate with CAPO-Si than BHA producing greater bone neoformation.

These findings are similar to those obtained by Shim et al. (2018) [53] who compared (in ARP technique) a test group using a synthetic hydroxyapatite combined with bone morphogenetic protein-2 with a control group using BHA alone. In biopsies, they harvested at three months, the percentage of neoformed bone in the test group was 25.37% compared with 6.13% in the control group, while the proportion of residual biomaterial was 12.03% in the test group compared with 16.79% in the control group.

A 90-day waiting period after ARP technique would appear insufficient to obtain complete resorption of the graft material in the alveolus and does not provide time to obtain greater bone neoformation with greater graft resorption. This was proved in a study by Ramaglia et al. (2018) [54] that compared two groups of alveoli treated with BHA, taking biopsies at different healing times (four and eight months). The histomorphometric results were better in the eight-month group, which presented a larger quantity of neoformed bone (35.58% vs. 47.76, respectively) and a lower proportion of residual biomaterial (34.23% vs. 25.43%, respectively).

In this way, it might be thought that longer healing periods would improve implant outcomes as a longer time would be provided for mineralization of the bone tissue in the alveolus after performing ARP technique. However, this idea has been belied by various systematic reviews such as those by Mardas et al. (2015) [55] and De Risi et al. (2015) [56]. These authors did not find significant differences in survival and success rates between studies that placed implants after different healing periods following ARP technique. According to this evidence, implant placement may be performed after a three- or four-month healing period, regardless of the bone graft material used.

## 5. Conclusions

Both the materials investigated presented similar behavior in terms of densitometric results, obtaining densities of over 1000 HU, CAPO-Si obtaining a higher density. Morphological findings revealed less horizontal bone loss in alveoli treated with CAPO-Si compared with those filled with BHA with statistically significant difference facilitating subsequent implant placement. ARP technique produced complete corticalization in 41.67% of alveoli treated with BHA but 58.34% of alveoli treated with CAPO-Si. Histomorphometric results showed greater bone neoformation and a smaller proportion of residual biomaterial in the CAPO-Si group compared with the BHA group, with statically significant differences showing that CAPO-Si could be a better biomaterial for bone regeneration. On the basis of the present findings, CAPO-Si obtains better results than BHA used for ARP technique. Additionally, this CAPO-Si material could be an alternative in some patients, for sociocultural and religious reasons due to its lower price and BHA animal origin.

## Figures and Tables

**Table 1:** Dimensional changes to test and control alveoli three months after dental extraction.

Alveolus	Horizontal Loss (mm) Mean ± SD	Vertical Loss (mm) Mean ± SD
Control (BHA)	1.3 ± 0.3	0.7 ± 0.2
Test (CAPO-Si)	0.99 ± 0.2	0.7 ± 0.3
*p*	0.017	0.636

**Table 2:** Distribution of the degree of corticalization obtained by control and test alveoli.

Patient	Control Corticalization	Test Corticalization
1	Partial	Complete
2	Partial	Partial
3	Complete	Partial
4	Absent	Absent
5	Complete	Complete
6	Partial	Absent
7	Absent	Complete
8	Complete	Complete
9	Partial	Partial
10	Complete	Complete
11	Partial	Complete
12	Complete	Complete

**Table 3:** Quantities of neoformed bone, lamellar bone, residual biomaterial, and connective tissue in test and control groups.

Alveolus	% of Neoformed Bone Mean ± SD	% of Lamellar Bone Mean ± SD	% of Residual Biomaterial Mean ± SD	% of Connective Tissue Mean ± SD
Control (BHA)	11 ± 7	9 ± 10	17 ± 13	62 ± 7
Test (CAPO-Si)	23 ± 15	14 ± 10	5 ± 10	58 ± 16
*p*	0.039	0.342	0.043	0.454

## Data Availability

The data presented in this study are available on request from the corresponding author.
